# The burden of neurological diseases in G7 countries from 1990 to 2021 and projections for the next 30 years: a Global Burden of Disease study

**DOI:** 10.3389/fpubh.2025.1632773

**Published:** 2026-01-29

**Authors:** Xiaohui Ji, Yanjun Lin, Xiangping Chen, Xuyang Jiang, Qiao Wang, Xiaoping Cui, Kuihua Wang

**Affiliations:** 1 Fuzong Clinical Medical College of Fujian Medical University, Fuzhou, China; 2 The 900th Hospital of Joint Logistic Support Force, PLA, Fuzhou, China

**Keywords:** neurological diseases, burden, G7 countries, epidemiological trend analysis, projection

## Abstract

**Background:**

Neurological disorders have become a significant global public health challenge due to their high rates of disability and mortality. This study analyzed epidemiological trends of neurological disorders in G7 countries from 1990 to 2021 based on Global Burden of Disease (GBD) 2021 data and predicted the disease burden for the next 30 years.

**Methods:**

Using the Joinpoint regression model and the Nordpred Age-Period-Cohort (APC) model, the study evaluated indicators such as incidence, mortality, and Disability-Adjusted Life Years (DALYs) of neurological disorders. It also analyzed the impact of gender, age, and social factors on the disease burden.

**Results:**

The overall burden of neurological disorders in G7 countries is lower than the global level, but there are significant gender differences. Women have a higher prevalence rate, which may be related to migraine and hormonal fluctuations, while men have more prominent Years of Life Lost (YLL) due to premature death, except in Japan and Italy. Regarding age distribution, the risk of disease gradually increases for individuals over 10 years old, and the mortality rate rises sharply after 70 years old. The association between aging and neurodegenerative diseases (such as Alzheimer’s disease) is particularly significant. Historical trend analysis from 1990 to 2021 shows that the global age-standardized incidence and mortality rates have remained stable overall. However, male mortality rates have increased significantly in the United States, Japan, and Germany. Predictions for the next 30 years indicate that despite stabilizing age-standardized rates, the number of neurological disease cases in G7 countries will continue to increase due to population growth and aging.

**Conclusion:**

The study untangles the unique challenges faced by G7 countries in preventing and controlling neurological disorders. It emphasizes the need to develop precise intervention strategies that consider age, gender, and social factors, providing valuable insights for developing countries.

## Introduction

The diseases affecting the nervous system can have lifelong impacts on patients, disrupting brain development and damaging the brain, spinal cord, or peripheral nerves (1). These conditions can compromise cognitive, sensory, socio-emotional, and motor functions and behaviors (2). This diverse group of neurological disorders includes congenital and neurodevelopmental disorders, cerebrovascular and neurodegenerative diseases, infections of the nervous system, neuro-immune diseases, neuromuscular or peripheral nervous system diseases, traumatic injuries, and cancers of the nervous system, collectively referred to as neurological diseases. These neurological diseases vary widely in their etiologies, symptoms, and disease progression (3). Among them, cerebrovascular diseases, neurodegenerative diseases, autoimmune diseases, spinal cord diseases, intracranial tumors, and craniocerebral trauma are the most common, causing significant impairment to cognitive functions and contributing to one of the highest global rates of morbidity, disability, and mortality. The outcomes of these diseases range from related functional impairments to severe lifelong disabilities or even death. While some of these conditions are treatable or preventable, others remain incurable. Over time, their epidemiological patterns have undergone significant changes, primarily due to population growth, aging, urbanization, and increased life expectancy, raising concerns about the increasing number of neurological disease cases and the associated global disease burden (4). More than 3.4 billion people worldwide are affected by neurological diseases, highlighting the urgent need for increased awareness and intervention (5). Studies from the Global Burden of Disease (GBD) 2021 database emphasize the significant morbidity and mortality associated with neurological diseases, underscoring the high disability-adjusted life years (DALYs) resulting from these conditions (6).

The extension of life expectancy can be regarded as one of the greatest achievements of healthcare systems worldwide (7). However, this increase has also led to a rise in age-related neurological diseases, such as Alzheimer’s disease and other dementias, strokes, and Parkinson’s disease. This shift demands that global health policies not only focus on survival but also prioritize minimizing health losses caused by disabilities by promoting functionality and independence. It’s important to note that not all neurological burdens are associated with population aging (8), making it crucial to quantify the overall health losses related to neurological conditions across the entire lifecycle (9).

Reducing the burden of neurological diseases is an integral part of the United Nations’ third Sustainable Development Goal, which aims to reduce premature deaths from chronic non-communicable diseases by one-third by 2030 (10). Due to continuous aging and increased life expectancy, a significant increase in the diagnosis of neurological diseases is expected in the coming decades. However, current research on the global burden of neurological diseases primarily focuses on global and developing country contexts, with limited studies in developed countries (11, 12). It is well-known that developed countries lead the world in neurological research and innovation, possessing advanced diagnostic techniques and therapies that significantly influence global medical advancements (13). The G7 member countries, as some of the most economically developed nations, have played a pivotal role in global economics, despite their share in the global economy declining somewhat due to the emergence of new economic powers (14). Nevertheless, they remain key economic players facing increasingly severe issues of population aging, leading to growing concerns about the rising number of neurological disease cases and the associated global disease burden.

To address the research gap regarding the burden of neurological diseases in developed countries, we aim to comprehensively examine the epidemiological trends of these diseases in major developed economies using the latest GBD 2021 dataset. Our focus will be on key indicators such as incidence, prevalence, mortality, and DALYs. By understanding these patterns, we can better inform public health strategies to reduce the burden of neurological diseases in these rapidly developing regions. Our findings will provide valuable insights to policymakers and healthcare professionals, supporting the development of more effective interventions and public health policies to manage and mitigate the global health impact of neurological diseases.

## Methods

The data used in this study comes from the GBD 2021 database, a broad and internationally recognized resource managed by the Institute for Health Metrics and Evaluation (IHME). GBD data is publicly available through IHME’s online platform.^1^ The database provides estimates of incidence, prevalence, mortality, and DALYs for various diseases, including neurological disorders. These data are standardized and adjusted for demographic factors, making them particularly suitable for cross-country comparisons like those in this study. The GBD study uses various data sources, including national surveys, hospital records, and vital registration systems, to ensure comprehensive and accurate estimates. It also incorporates advanced statistical modeling techniques, such as Bayesian methods, to account for uncertainty and generate 95% uncertainty intervals (UIs) for all estimates. The rigorous methodology and broad coverage of the GBD database make it one of the most reliable sources for analyzing global health trends.

### Joinpoint regression analysis

To assess trends over time, we used joinpoint regression analysis with the Joinpoint software version 5.0. This method identifies points where trends undergo significant changes and calculates the annual percentage change (APC) for each time segment. We used joinpoint regression to analyze trends and identify specific periods when significant changes occurred in neurological disease indicators for each country. These indicators provided detailed insights into the changing epidemiological burden among G7 member countries. Besides APC, age-standardized incidence rates were also calculated for each country. We also compared DALYs rates between males and females to evaluate gender differences in the burden of neurological diseases. Data were analyzed with 95% UIs to account for variability and estimate the precision of the results.

### Prediction model

To reflect trends in the burden of neurological diseases, the Nordpred APC analysis was used to predict the number of new cases of neurological diseases from 2022 to 2042 by gender. The R software (R 4.3.1) was utilized for this analysis, which considered the rate of change and varying population structures, as demonstrated in previous study (15). Additionally, to facilitate comparison with the prediction results, we calculated the absolute number of events that would occur if the rates remained stable (baseline reference), decreased by 1% per year (optimistic reference), or increased by 1% per year (pessimistic reference) based on the actual observed rates in 2019.

## Result

### Descriptive analysis

Table 1 presents the global and national epidemiological burden of neurological diseases, detailing metrics including prevalence, incidence, mortality, Disability-Adjusted Life Years (DALYs), Years Lived with Disability (YLDs), and Years of Life Lost (YLLs). The data reveal a substantial global burden, with an estimated 2.87 billion prevalent cases, 823.8 million incident cases, and 2.61 million deaths annually, culminating in 112.0 million DALYs. Cross-national comparisons of age-standardized rates highlight significant variation, with the United States, Italy, and Germany exhibiting a higher overall burden of neurological diseases, whereas Japan reported the lowest rates across key metrics. A consistent sex disparity was observed, wherein females had a higher prevalence of neurological disorders globally and in most nations, while males generally endured a greater burden from premature mortality, as evidenced by higher YLLs. These findings underscore the profound and varied public health impact of neurological diseases across different populations.

**Table 1:** Global and G7 countries’ all-age cases and age-standardized prevalence, incidence, deaths, YLLs, YLDs, and DALYs rates of neurological disorders in 2021.

Nation	Measure		Prevalence	Incidence	Deaths	Dalys^*^	Ylds^*^	Ylls^*^
Global	All-Ages Cases	Total	2870478131 (2664843919,3088261583)	823798510 (733108418,910525551)	2607635 (1193364,5592301)	112028844 (65444986,178637758)	71065533 (32123543,128889654)	40963311 (23021323,79408074)
		Male	1301358647 (1197752037,1411650278)	390143579 (345431305,432154921)	995849 (535964,2030053)	46414695 (28687819,70731080)	28459906 (13714449,50430969)	17954789 (11645135,32251868)
		Female	1569119484 (1459513915,1681656611)	433654931 (387393796,477930524)	1611786 (661865,3577642)	65614149 (36152258,107057212)	42605627 (18365223,77873502)	23008522 (11378849,47605186)
	Age-Standardized Rates Per 100000 People	Total	35333.86 (32764.82,38056.12)	10264.27 (9137.9,11349.79)	33.22 (15.07,71.7)	1385.1 (807.66,2209.43)	874.97 (394.86,1586.53)	510.13 (286.6,984.59)
		Male	32101.61 (29574.78,34850.29)	9706.63 (8598.16,10757.26)	31.09 (15.93,65.16)	1224.86 (768.59,1903.06)	719.65 (354.89,1260.53)	505.21 (314.95,933.24)
		Female	38563.4 (35846.19,41361.49)	10825.19 (9666.52,11941.37)	34.2 (14.22,75.58)	1528.91 (833.67,2529.89)	1024.56 (425.17,1896.76)	504.35 (259.77,1026.89)
Canada	All-Ages Cases	Total	17508183 (16076255,19018838)	4818978 (4314180,5346804)	26175 (12910,53863)	827569 (525257,1271246)	469791 (250707,790753)	357777 (202060,675411)
		Male	7494375 (6785891,8234327)	2211324 (1967930,2463318)	10626 (6039,20548)	321061 (223319,466081)	161648 (94192,257702)	159413 (102470,279549)
		Female	10013808 (9259075,10810128)	2607654 (2326831,2889130)	15549 (6814,33295)	506508 (298419,807765)	308143 (151314,530997)	198365 (100828,395862)
	Age-Standardized Rates Per 100000 People	Total	43216.52 (39794.76,46954.75)	12597.52 (11133.37,14117.61)	31.41 (16.29,63.21)	1476.49 (849.29,2349.95)	984.1 (440.93,1796.22)	492.38 (308.46,864.05)
		Male	37539.26 (33797.99,41325.1)	11634.35 (10284.53,13068.5)	31.96 (18.33,60.97)	1201.44 (812.07,1799.44)	684.54 (358.13,1169.89)	516.9 (351.07,862.66)
		Female	48886.47 (45143.73,52703.59)	13576.34 (12022.9,15199.7)	30.76 (14.63,64.2)	1746.66 (887.03,2944.82)	1279.1 (494.93,2396.37)	467.56 (271.8,859.07)
Italy	All-Ages Cases	Total	29752417 (27783736,31590619)	8300907 (7414081,9122534)	79323 (33598,169369)	1833632 (1119511,2979303)	885203 (474728,1481447)	948429 (444267,1961111)
		Male	13119121 (12109637,14035913)	3843603 (3417153,4265681)	25996 (12980,54866)	648423 (413573,1030468)	299901 (161391,506821)	348522 (194115,695417)
		Female	16633296 (15589648,17663204)	4457304 (3979844,4891046)	53327 (20498,115375)	1185209 (703064,1960437)	585302 (314511,983093)	599907 (248933,1265694)
	Age-Standardized Rates Per 100000 People	Total	45388.35 (42347.16,48368.85)	13598.34 (12074.83,15047.15)	38.67 (17.59,80.82)	1610.58 (860.69,2656.92)	1060.48 (412.84,2011.96)	550.1 (301.02,1040.93)
		Male	41155.54 (38060.27,44319.79)	12873.41 (11313.06,14335.57)	35.49 (18.38,73.2)	1305.98 (767.05,2060.78)	769.36 (325.73,1449.21)	536.62 (329.6,986.31)
		Female	49655.2 (46456.1,52667.26)	14335.98 (12786.29,15778.39)	39.89 (16.53,84.38)	1892.76 (939.46,3249.77)	1346.3 (478.08,2576.68)	546.46 (270.71,1063.35)
United States Of America	All-Ages Cases	Total	158831484 (148028213,169350047)	46398332 (41399776,51216197)	261531 (120299,550300)	7469285 (4623090,11694897)	3934667 (2026448,6851624)	3534617 (1844515,6942515)
		Male	69291993 (63936949,74953305)	21207657 (18671375,23539748)	101722 (54489,206041)	2890490 (1917983,4385820)	1390246 (768903,2344399)	1500245 (908224,2773090)
		Female	89539492 (83609284,95181168)	25190674 (22527729,27741040)	159809 (65752,344794)	4578794 (2690132,7381426)	2544422 (1230308,4478361)	2034373 (940004,4182373)
	Age-Standardized Rates Per 100000 People	Total	44520.72 (41569.49,47744.59)	13627.32 (12080.74,15062.47)	39.9 (19.01,83.13)	1573.07 (896.68,2501.79)	984.08 (435.38,1811.98)	588.99 (331.62,1107.73)
		Male	39584.92 (36539.64,42972.32)	12610.9 (11056.83,14082.91)	39.76 (21.35,80.04)	1326.44 (859.33,1990.02)	723.74 (376.78,1266.02)	602.69 (377.11,1085.63)
		Female	49407.4 (46095.81,52673.57)	14646.85 (13084.87,16205.09)	39.81 (17.2,85.18)	1810.98 (946.98,2992.56)	1237.41 (483.69,2324.68)	573.57 (294.53,1127.06)
France	All-Ages Cases	Total	30005068 (27699786,32388951)	8350268 (7464148,9194492)	61896 (28256,129953)	1611911 (1015321,2525985)	831893 (432227,1416996)	780018 (414795,1520182)
		Male	13428243 (12153051,14684138)	3859260 (3431711,4284450)	22295 (12154,44963)	621407 (417464,946086)	303046 (156693,523972)	318361 (200805,576663)
		Female	16576824 (15381824,17854661)	4491008 (4015003,4926526)	39601 (16289,85037)	990504 (595839,1598664)	528847 (273478,902994)	461657 (218762,941211)
	Age-Standardized Rates Per 100000 People	Total	42733.14 (39295.14,46317.03)	12446.4 (10969.1,13935.54)	31.86 (16.23,63.58)	1516.82 (865.16,2452.29)	1009.54 (432.43,1875.89)	507.28 (319.39,876.08)
		Male	39472.97 (35555.77,43199.91)	11845.7 (10407.87,13259.73)	32.5 (18.65,63.43)	1320.18 (833.62,2027.78)	779.22 (347.88,1443.92)	540.96 (374.54,897.88)
		Female	45924.98 (42513.48,49447.64)	13039.18 (11540.42,14577.04)	30.9 (14.57,62.87)	1705.33 (900.58,2826.63)	1232.17 (489.9,2321.71)	473.17 (273.74,850.39)
Japan	All-Ages Cases	Total	52495557 (48488917,56223478)	15574152 (13918280,17116228)	202194 (82335,423421)	3786763 (2328366,6156542)	1509714 (953480,2310284)	2277048 (1004240,4664279)
		Male	22649610 (20704531,24540950)	6958618 (6137518,7669928)	61902 (28983,133211)	1312048 (854120,2133946)	519176 (326041,793910)	792871 (410337,1627258)
		Female	29845947 (27666171,31813049)	8615534 (7736866,9507611)	140292 (53036,292254)	2474715 (1466282,4053178)	990538 (615816,1516898)	1484177 (594210,3047238)
	Age-Standardized Rates Per 100000 People	Total	37095.31 (34384.49,40229.29)	11769.66 (10434.33,13007.24)	33.29 (14.79,68.22)	1183.86 (724.15,1838.74)	708.94 (345.25,1267.51)	474.92 (251.1,890.16)
		Male	32971.94 (30158.64,36158.72)	10778.55 (9472.47,11945.38)	29.79 (14.87,62.35)	1007.76 (660.01,1510.66)	548.24 (297.75,954.76)	459.52 (273.58,851.07)
		Female	41281.45 (38351.2,44502.08)	12780.95 (11319.38,14145.3)	34.67 (14.23,70.65)	1335.25 (763.33,2156.66)	863.64 (387.82,1578.17)	471.61 (226.47,902.88)
Germany	All-Ages Cases	Total	42036863 (39136048,45198397)	11018248 (9792509,12292860)	100351 (44477,206037)	2591625 (1619548,4097332)	1333375 (730840,2256061)	1258250 (633302,2447334)
		Male	18751146 (17160621,20432305)	5223758 (4586745,5797242)	35008 (18683,70857)	980709 (644601,1476954)	486233 (276393,812691)	494476 (296901,920967)
		Female	23285717 (21682370,24893233)	5794489 (5175554,6512764)	65343 (25794,136232)	1610916 (973990,2636602)	847143 (456059,1412348)	763774 (336007,1549457)
	Age-Standardized Rates Per 100000 People	Total	45204 (41754.06,48657.85)	12694.8 (11145.36,14190.94)	39.65 (18.8,78.85)	1728.79 (966.55,2778.46)	1148.13 (501.31,2165.06)	580.67 (337.62,1053.28)
		Male	40417.52 (36785.26,44220.99)	11988.51 (10476.72,13472.08)	36.83 (20.04,73.09)	1443.19 (892.64,2231.7)	867.21 (415.69,1593.94)	575.98 (372.62,1002.95)
		Female	50263.04 (46765.52,53690.31)	13443.36 (11869.98,15114.08)	40.43 (17.41,82.52)	2008.11 (1051.91,3386.93)	1440.17 (569.98,2702.2)	567.94 (298.47,1075.89)
United Kingdom	All-Ages Cases	Total	32162946 (29893384,34352391)	9281803 (8289803,10268223)	53058 (25879,108805)	1565821 (992464,2419383)	837239 (421754,1448790)	728582 (420569,1386347)
		Male	14230992 (13069027,15356827)	4294522 (3829358,4751000)	21065 (12136,40800)	621417 (424239,917033)	299764 (158378,510293)	321654 (212259,567737)
		Female	17931953 (16835179,19053755)	4987281 (4464357,5491243)	31992 (13798,68580)	944403 (566156,1500808)	537475 (266525,940581)	406928 (206609,816772)
	Age-Standardized Rates Per 100000 People	Total	44279.64 (41269.92,47359.89)	13403.47 (11923.82,14863.22)	34.34 (17.96,68.3)	1575.6 (913.11,2497.13)	1017.25 (431.42,1907.88)	558.35 (364.06,965.31)
		Male	40066.2 (36914.16,43390.74)	12639.65 (11196.42,14074.97)	33.58 (19.88,63.54)	1332.94 (859.54,2014.03)	752.59 (345.6,1352.39)	580.35 (412.22,952.35)
		Female	48388.78 (45311.62,51402.01)	14156.75 (12583.23,15655)	34.28 (16.24,70.82)	1801.48 (972.92,2970.85)	1271.38 (498.6,2429.53)	530.09 (316.44,961.35)

* DALYs, disability-adjusted life-years; YLDs, years lived with disability; YLLs, years of life lost.

**Figure 1:**
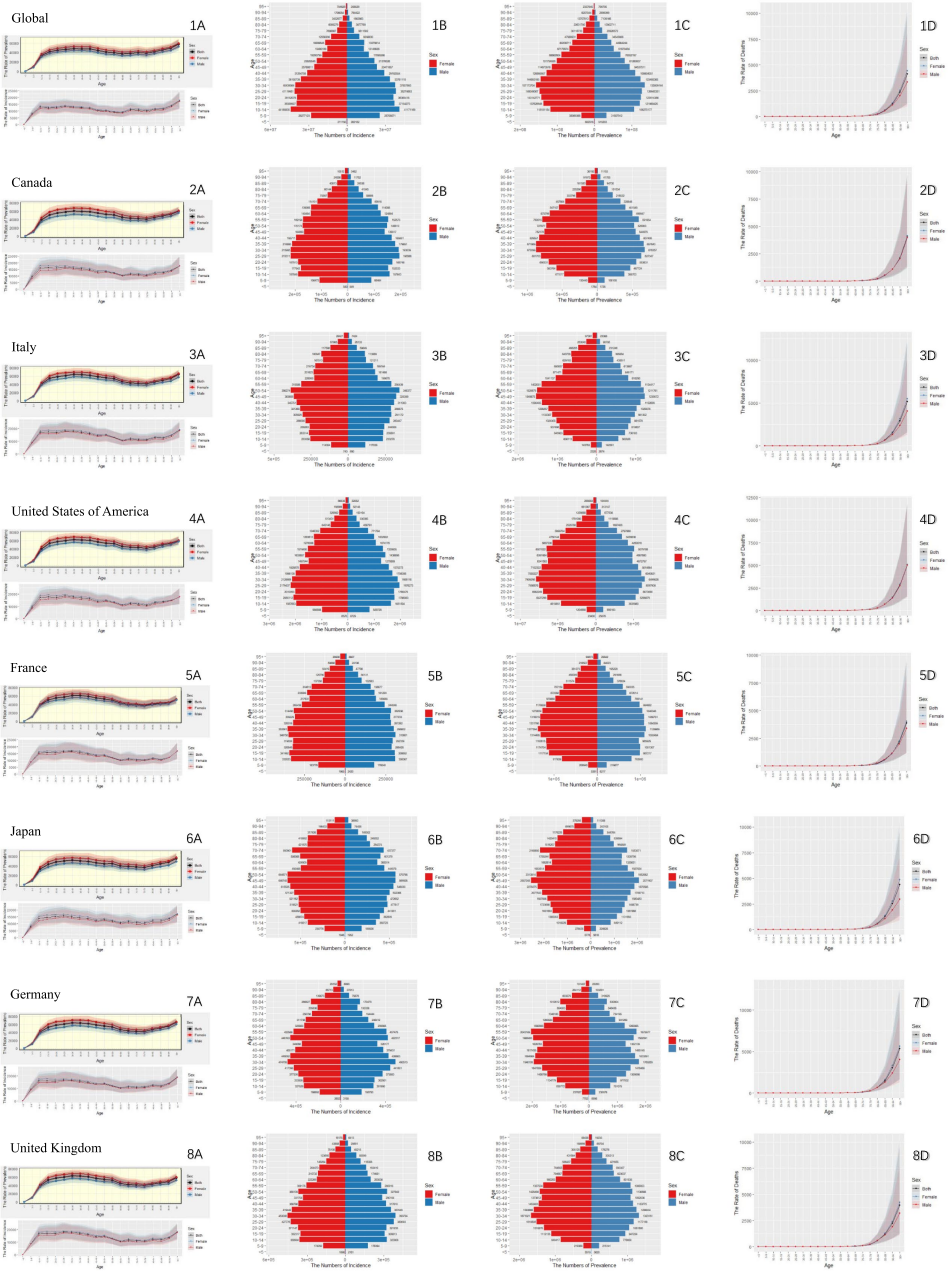
Age-specific numbers and age-standardized prevalence, incidence, and mortality rates of neurological disorders globally and in G7 countries in 2021. **(A)** Line graph depicting the prevalence and incidence rates across different age groups. **(B)** Incidence-based population pyramid across different age groups. **(C)** Pyramid of prevalence across different age groups. **(D)** Line graph showing mortality rates across different age groups.

As delineated in Figure 1, our comprehensive analysis of neurological disorders across global and G7 populations reveals a consistent epidemiological triad: disease burden demonstrates a powerful age-dependent escalation, peaking in the oldest age groups, while exhibiting a critical sex dissociation—females bear a higher prevalence, indicating greater accumulated lifetime burden, whereas males systematically experience higher incidence and mortality, suggesting increased susceptibility or fatality.

**Figure 2:**
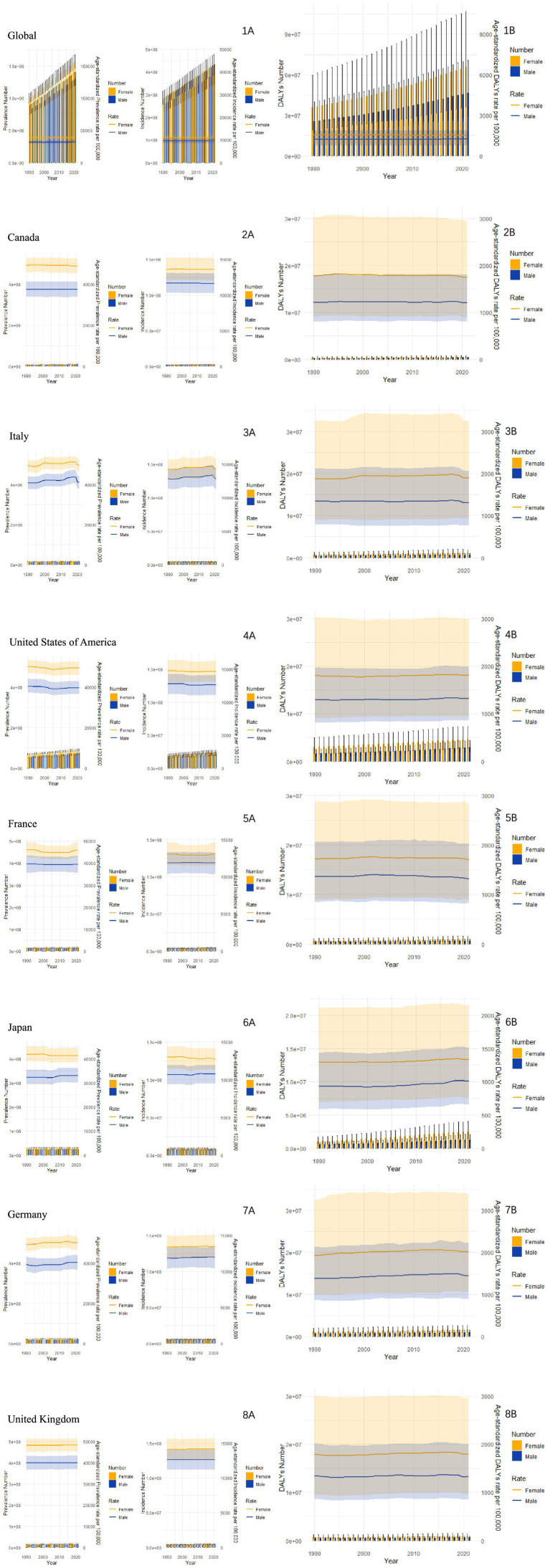
Trends in all-age cases and age-standardized incidence, mortality, and DALY rates of neurological disorders by sex from 1990 to 2021. **(A)** Trends in the prevalence and incidence of neurological diseases in different years. **(B)** Trends in the DALYs of neurological diseases in different years.

**Figure 3:**
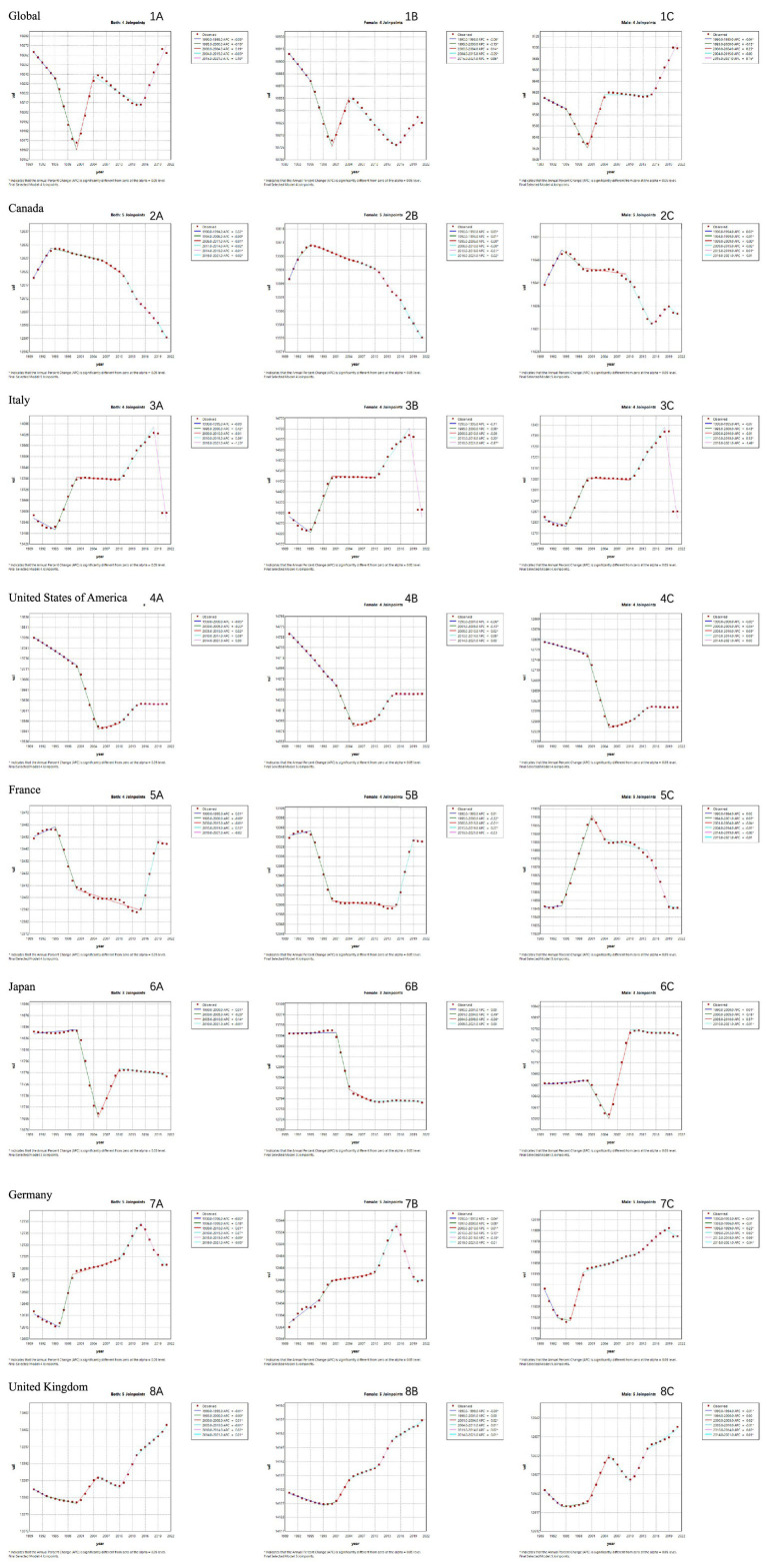
Joinpoint regression analysis of sex-specific age-standardized incidence rates of neurological disorders globally and in G7 countries from 1990 to 2021. **(A)** Joinpoint regression analysis of age-standardized incidence rates (ASIR) across genders. **(B)** Joinpoint regression analysis of age-standardized incidence rates in females. **(C)** Joinpoint regression analysis of age-standardized incidence rates in females.

**Figure 4:**
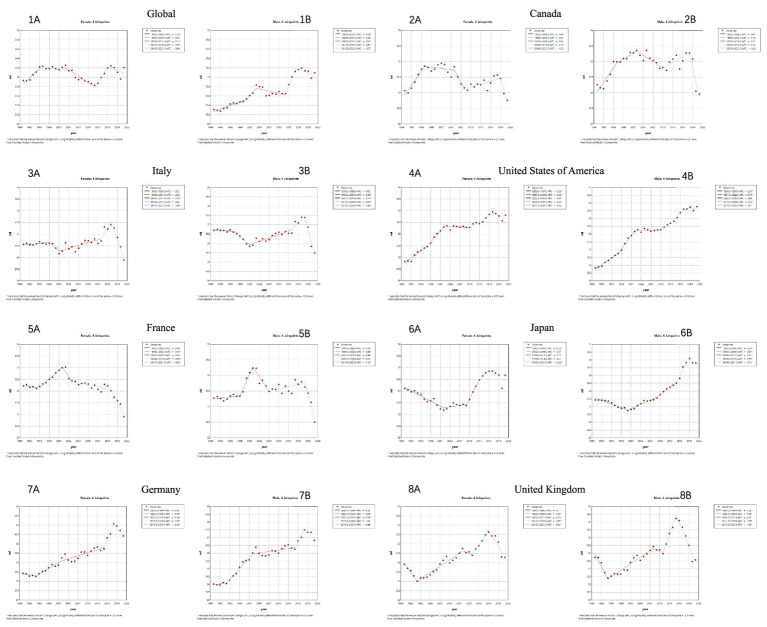
Joinpoint regression analysis of sex-specific age-standardized mortality rates of neurological disorders globally and in G7 countries from 1990 to 2021. **(A)** Joinpoint regression analysis of age-standardized deaths in females. **(B)** Joinpoint regression analysis of age-standardized deaths in males.

In comparison to global statistics, the G7 countries exhibit a noteworthy reduction in the burden of neurological diseases. Figure 2 outlines the trends in gender-specific incidence and mortality rates of nervous system diseases from 1990 to 2021. While these rates fluctuate slightly over the years, it’s apparent that the mortality rate for women surpasses that of men in various regions. The age-standardized DALY remains stable for both genders, although there’s a gradual increase in Japan and the US (Figures 2-4B,6B).

Joinpoint regression analysis, depicted in Figure 3, reveals intriguing trends in the age-standardized incidence rate of neurological diseases from 1990 to 2021. Notably, some countries deviate from the global trend. For instance, from 1990 to 1999, the global incidence rate for both genders declined. However, apart from the US, Japan, and the UK (Figures 3-4A,6A,8A), other G7 nations experienced an upward trajectory. Since 2015, the global incidence rate has been on the rise for both genders, whereas the G7 countries show a contrasting trend.

From 1990 to 2021, in terms of global trends, the age-standardized mortality rate for males showed an upward trend, while that for females remained largely unchanged (Figures 4-1A,1B). However, the age-standardized mortality rates for males in the United States, Japan, and Germany (Figures 4-4B,6B,7B) did not improve as we had anticipated, but followed the same trend. Even the increase in age-standardized mortality rates for females in the United States and Germany (Figures 4-4A,7A) was greater than the global level.

Table 2 presents the AAPC of incidence and mortality rates for neurological diseases over more than three decades. From 1990 to 2021, the age-standardized incidence rate (0.00 (−0.00 to 0.01)) and mortality rate (0.02 (−0.02 to 0.06)) of neurological diseases globally remained stable. The relevant data within the G7 member countries were roughly the same. However, the age-standardized mortality rates in the United States, France, Japan, and Germany (0.02 (−0.02 to 0.06)) increased significantly. Surprisingly, the AAPC of mortality rates for males in the United States and Japan was much higher than that for females (Japan 0.26 (0.16–0.35), United States 0.26 (0.16–0.35)).

**Table 2:** Joinpoint regression analysis of sex-specific age-standardized incidence and mortality rates (per 100,000) of neurological disorders globally and in G7 countries from 1990 to 2021.

Nation	Both								Female						Male				
Global	ASIR	Period	1990-1995	1995-2000	2000-2004	2004-2015	2015-2021		1990-1995	1995-2000	2000-2004	2004-2015	2015-2021		1990-1995	1995-2000	2000-2004	2004-2015	2015-2021
		APC	-0.05 (-0.07 - -0.04)	-0.15 (-0.17 - -0.12)	0.19 (0.15 - 0.23)	-0.03 (-0.03 - -0.02)	0.10 (0.09 - 0.12)		-0.06 (-0.08 – -0.05)	-0.15 (-0.17 - -0.13)	0.14 (0.11 - 0.17)	-0.05 (-0.06 - -0.05)	0.06 (0.04 - 0.07)		-0.04 (-0.06 - -0.02)	-0.15 (-0.18 - -0.12)	0.25 (0.20 - 0.30)	-0.00 (-0.01 - 0.01)	0.16 (0.14 - 0.17)
		AAPC	0.00 (-0.00 - 0.01)						-0.02 (-0.03 – -0.02)						0.03 (0.02 - 0.04)				
	ASMR	Period	1990-2003	2003-2012	2012-2016	2016-2021			1990-1999	1999-2012	2012-2017	2017-2021			1990-2000	2000-2003	2003-2007	2007-2012	2012-2015
		APC	0.07 (0.04 - 0.10)	-0.12 (-0.18 - -0.07)	0.34 (0.09 - 0.60)	-0.10 (-0.22 - 0.01)			0.12 (0.06 - 0.17)	-0.10 (-0.14 - -0.07)	0.23 (0.05 - 0.42)	-0.11 (-0.29 - 0.07)			0.08 (0.05 - 0.11)	0.34 (-0.08 - 0.75)	-0.17 (-0.37 - 0.04)	0.03 (-0.10 - 0.16)	0.54 (0.14 - 0.95)
		AAPC	0.02 (-0.02 - 0.06)						0.01 (-0.03 - 0.06)						0.08 (0.02 - 0.15)				
Canada	ASIR	Period	1990-1994	1994-2006	2006-2011	2011-2014	2014-2019	2019-2021	1990-1992	1992-1995	1995-2010	2010-2016	2016-2021		1990-1994	1994-1999	1999-2009	2009-2015	2015-2019
		APC	0.02 (0.02 - 0.02)	-0.00 (-0.00 - -0.00)	-0.01 (-0.01 - -0.01)	-0.02 (-0.03 - -0.02)	-0.01 (-0.02 - -0.01)	-0.02 (-0.03 - -0.02)	0.03 (0.02 - 0.03)	0.01 (0.01 - 0.02)	-0.00 (-0.00 - -0.00)	-0.01 (-0.02 - -0.01)	-0.02 (-0.02 - -0.02)		0.02 (0.01 - 0.02)	-0.01 (-0.01 - -0.00)	-0.00 (-0.00 - -0.00)	-0.02 (-0.02 - -0.01)	0.01 (0.00 - 0.01)
		AAPC	-0.01 (-0.01 - -0.01)						-0.01 (-0.01 - -0.00)						-0.00 (-0.00 - -0.00)				
	ASMR	Period	1990-2000	2000-2009	2009-2019	2019-2021			1990-1996	1996-2005	2005-2008	2008-2019	2019-2021		1990-2000	2000-2011	2011-2018	2018-2021	
		APC	0.32 (0.22 - 0.43)	-0.23 (-0.37 - -0.09)	0.09 (-0.03 - 0.20)	-1.61 (-2.82 - -0.39)			0.49 (0.26 - 0.71)	-0.04 (-0.18 - 0.09)	-0.65 (-1.88 - 0.61)	0.10 (0.01 - 0.20)	-1.23 (-2.42 - -0.02)		0.38 (0.26 - 0.51)	-0.15 (-0.27 - -0.03)	0.20 (-0.05 - 0.45)	-1.44 (-2.16 - -0.71)	
		AAPC	-0.04 (-0.14 - 0.06)						-0.02 (-0.17 - 0.12)						-0.02 (-0.13 - 0.08)				
Italy	ASIR	Period	1990-1995	1995-2000	2000-2010	2010-2018	2018-2021		1990-1995	1995-2000	2000-2010	2010-2018	2018-2021		1990-1995	1995-2000	2000-2010	2010-2018	2018-2021
		APC	-0.09 (-0.30 - 0.12)	0.42 (0.13 - 0.72)	-0.01 (-0.09 - 0.07)	0.26 (0.14 - 0.38)	-1.23 (-1.69 - -0.77)		-0.11 (-0.27 - 0.06)	0.38 (0.14 - 0.61)	-0.00 (-0.07 - 0.06)	0.20 (0.11 - 0.30)	-0.97 (-1.33 - -0.62)		-0.07 (-0.32 - 0.18)	0.48 (0.13 - 0.84)	-0.01 (-0.11 - 0.09)	0.32 (0.17 - 0.48)	-1.49 (-2.05 - -0.92)
		AAPC	-0.00 (-0.08 - 0.08)						-0.00 (-0.06 - 0.06)						0.00 (-0.09 - 0.10)				
	ASMR	Period	1990-2006	2006-2018	2018-2021				1990-2006	2006-2018	2018-2021				1990-1996	1996-2001	2001-2014	2014-2018	2018-2021
		APC	-0.08 (-0.12 - -0.03)	0.21 (0.13 - 0.29)	-1.34 (-1.88 - -0.80)				-0.05 (-0.09 – -0.00)	0.20 (0.13 - 0.27)	-1.13 (-1.63 - -0.62)				-0.03 (-0.20 - 0.13)	-0.40 (-0.71 - -0.09)	0.17 (0.11 - 0.23)	0.51 (0.05 - 0.98)	-1.92 (-2.37 - -1.48)
		AAPC	-0.09 (-0.15 - -0.03)						-0.06 (-0.11 - 0.00)						-0.12 (-0.21 - -0.03)				
United States of America	ASIR	Period	1990-2000	2000-2005	2005-2010	2010-2014	2014-2021		1990-2001	2001-2004	2004-2007	2007-2011	2011-2014	2014-2021	1990-2000	2000-2005	2005-2010	2010-2014	2014-2021
		APC	-0.05 (-0.05 - -0.05)	-0.22 (-0.23 - -0.22)	0.02 (0.01 - 0.03)	0.08 (0.07 - 0.09)	0.00 (-0.00 - 0.01)		-0.06 (-0.06 – -0.06)	-0.15 (-0.18 - -0.13)	-0.03 (-0.05 - -0.00)	0.03 (0.02 - 0.05)	0.09 (0.07 - 0.12)	0.00 (-0.00 - 0.00)	-0.03 (-0.03 - -0.03)	-0.34 (-0.35 - -0.33)	0.03 (0.02 - 0.04)	0.08 (0.07 - 0.10)	0.00 (-0.00 - 0.00)
		AAPC	-0.04 (-0.04 - -0.04)						-0.03 (-0.03 – -0.02)						-0.05 (-0.05 - -0.05)				
	ASMR	Period	1990-2001	2001-2009	2009-2017	2017-2021			1990-2002	2002-2009	2009-2015	2015-2021			1990-2001	2001-2015	2015-2018	2018-2021	
		APC	-0.20 (-0.27 - -0.13)	0.16 (0.03 - 0.29)	0.58 (0.46 - 0.71)	-0.19 (-0.47 - 0.09)			-0.22 (-0.29 – -0.15)	0.08 (-0.12 - 0.27)	0.71 (0.46 - 0.97)	-0.22 (-0.40 - -0.04)			-0.25 (-0.32 - -0.19)	0.44 (0.39 - 0.49)	1.75 (0.84 - 2.66)	-0.17 (-0.60 - 0.27)	
		AAPC	0.10 (0.04 - 0.16)						0.03 (-0.05 - 0.10)						0.26 (0.16 - 0.35)				
France	ASIR	Period	1990-1995	1995-2000	2000-2015	2015-2019	2019-2021		1990-1995	1995-2000	2000-2015	2015-2019	2019-2021		1990-1994	1994-2001	2001-2004	2004-2014	2014-2019
		APC	0.01 (0.00 - 0.02)	-0.08 (-0.10 - -0.07)	-0.01 (-0.01 - -0.01)	0.12 (0.10 - 0.14)	-0.02 (-0.06 - 0.03)		0.01 (-0.00 - 0.03)	-0.22 (-0.24 - -0.20)	-0.01 (-0.01 - -0.00)	0.27 (0.24 - 0.31)	-0.03 (-0.10 - 0.04)		0.00 (-0.01 - 0.01)	0.07 (0.06 - 0.07)	-0.04 (-0.07 - -0.01)	-0.01 (-0.01 - -0.00)	-0.06 (-0.07 - -0.05)
		AAPC	-0.00 (-0.01 - 0.00)						-0.00 (-0.01 - 0.00)						-0.00 (-0.00 - 0.00)				
	ASMR	Period	1990-1997	1997-2002	2002-2007	2007-2017	2017-2021		1990-1995	1995-2002	2002-2005	2005-2017	2017-2021		1990-1998	1998-2002	2002-2007	2007-2018	2018-2021
		APC	0.00 (-0.16 - 0.16)	0.49 (0.13 - 0.86)	-0.47 (-0.82 - -0.12)	-0.01 (-0.11 - 0.09)	-0.83 (-1.18 - -0.49)		-0.06 (-0.28 - 0.16)	0.40 (0.23 - 0.56)	-0.65 (-1.58 - 0.29)	-0.09 (-0.15 - -0.03)	-0.82 (-1.10 - -0.54)		0.04 (-0.09 - 0.16)	0.69 (0.14 - 1.24)	-0.46 (-0.80 - -0.13)	0.07 (-0.02 - 0.15)	-1.12 (-1.67 - -0.56)
		AAPC	-0.11 (-0.21 - -0.01)						-0.12 (-0.23 - -0.02)						-0.06 (-0.17 - 0.04)				
Japan	ASIR	Period	1990-2000	2000-2005	2005-2010	2010-2021			1990-2001	2001-2004	2004-2009	2009-2021			1990-2000	2000-2005	2005-2010	2010-2021	
		APC	0.01 (0.00 - 0.01)	-0.26 (-0.27 - -0.25)	0.14 (0.13 - 0.16)	-0.01 (-0.01 - -0.01)			0.00 (-0.00 - 0.00)	-0.49 (-0.53 - -0.45)	-0.06 (-0.07 - -0.05)	0.00 (-0.00 - 0.00)			0.01 (0.00 - 0.01)	-0.16 (-0.17 - -0.15)	0.37 (0.36 - 0.38)	-0.01 (-0.01 - -0.01)	
		AAPC	-0.02 (-0.02 - -0.02)						-0.06 (-0.06 – -0.05)						0.03 (0.03 - 0.04)				
	ASMR	Period	1990-2001	2001-2009	2009-2017	2017-2021			1990-2002	2002-2009	2009-2015	2015-2021			1990-2001	2001-2015	2015-2018	2018-2021	
		APC	-0.20 (-0.27 - -0.13)	0.16 (0.03 - 0.29)	0.58 (0.46 - 0.71)	-0.19 (-0.47 - 0.09)			-0.22 (-0.29 – -0.15)	0.08 (-0.12 - 0.27)	0.71 (0.46 - 0.97)	-0.22 (-0.40 - -0.04)			-0.25 (-0.32 - -0.19)	0.44 (0.39 - 0.49)	1.75 (0.84 - 2.66)	-0.17 (-0.60 - 0.27)	
		AAPC	0.10 (0.04 - 0.16)						0.03 (-0.05 - 0.10)						0.26 (0.16 - 0.35)				
Germany	ASIR	Period	1990-1996	1996-1999	1999-2010	2010-2015	2015-2021		1990-1997	1997-2000	2000-2010	2010-2015	2015-2019	2019-2021	1990-1993	1993-1996	1996-1999	1999-2012	2012-2018
		APC	-0.02 (-0.03 - -0.01)	0.18 (0.13 - 0.23)	0.01 (0.01 - 0.02)	0.07 (0.05 - 0.09)	-0.08 (-0.08 - -0.07)		0.04 (0.03 - 0.05)	0.08 (0.00 - 0.16)	0.01 (0.00 - 0.02)	0.13 (0.10 - 0.15)	-0.18 (-0.22 - -0.14)	-0.01 (-0.09 - 0.07)	-0.14 (-0.17 - -0.12)	-0.01 (-0.07 - 0.04)	0.25 (0.20 - 0.30)	0.02 (0.02 - 0.03)	0.06 (0.05 - 0.07)
		AAPC	0.01 (0.01 - 0.02)						0.02 (0.01 - 0.03)						0.03 (0.02 - 0.03)				
	ASMR	Period	1990-1994	1994-2002	2002-2015	2015-2018	2018-2021		1990-2015	2015-2018	2018-2021				1990-1994	1994-2003	2003-2006	2006-2015	2015-2018
		APC	0.01 (-0.37 - 0.38)	0.39 (0.24 - 0.54)	0.08 (0.01 - 0.14)	1.00 (-0.06 - 2.06)	-0.61 (-1.12 - -0.09)		0.17 (0.15 - 0.19)	1.01 (-0.07 - 2.10)	-0.48 (-1.01 - 0.05)				0.07 (-0.30 - 0.43)	0.68 (0.56 - 0.80)	-0.34 (-1.38 - 0.71)	0.19 (0.07 - 0.30)	0.91 (-0.14 - 1.97)
		AAPC	0.17 (0.05 - 0.29)						0.19 (0.07 - 0.30)						0.27 (0.12 - 0.43)				
United kingdom	ASIR	Period	1990-2000	2000-2005	2005-2010	2010-2014	2014-2021		1990-1998	1998-2001	2001-2004	2004-2011	2011-2014	2014-2021	1990-1994	1994-2000	2000-2005	2005-2010	2010-2014
		APC	-0.00 (-0.00 - -0.00)	0.01 (0.01 - 0.01)	-0.01 (-0.01 - -0.00)	0.02 (0.01 - 0.02)	0.01 (0.01 - 0.01)		-0.00 (-0.00 - -0.00)	0.00 (-0.00 - 0.01)	0.02 (0.01 - 0.02)	0.00 (0.00 - 0.01)	0.02 (0.02 - 0.03)	0.01 (0.01 - 0.01)	-0.01 (-0.01 - -0.01)	0.00 (-0.00 - 0.00)	0.02 (0.02 - 0.02)	-0.01 (-0.01 - -0.01)	0.02 (0.01 - 0.02)
		AAPC	0.00 (0.00 - 0.01)						0.01 (0.01 - 0.01)						0.00 (0.00 - 0.00)				
	ASMR	Period	1990-1994	1994-2008	2008-2011	2011-2016	2016-2021		1990-1994	1994-2008	2008-2011	2011-2016	2016-2021		1990-1994	1994-2008	2008-2011	2011-2015	2015-2021
		APC	-0.84 (-1.30 - -0.38)	0.34 (0.27 - 0.42)	-0.29 (-1.62 - 1.06)	0.93 (0.50 - 1.36)	-1.14 (-1.43 - -0.85)		-0.71 (-1.16 - -0.26)	0.34 (0.27 - 0.42)	-0.20 (-1.50 - 1.12)	0.77 (0.35 - 1.19)	-0.84 (-1.12 - -0.55)		-1.22 (-1.79 - -0.64)	0.42 (0.33 - 0.52)	-0.31 (-1.99 - 1.41)	1.76 (0.88 - 2.65)	-1.43 (-1.71 - -1.15)
		AAPC	-0.02 (-0.18 - 0.14)						0.03 (-0.12 - 0.19)						-0.05 (-0.26 - 0.16)				

**Figure 5:**
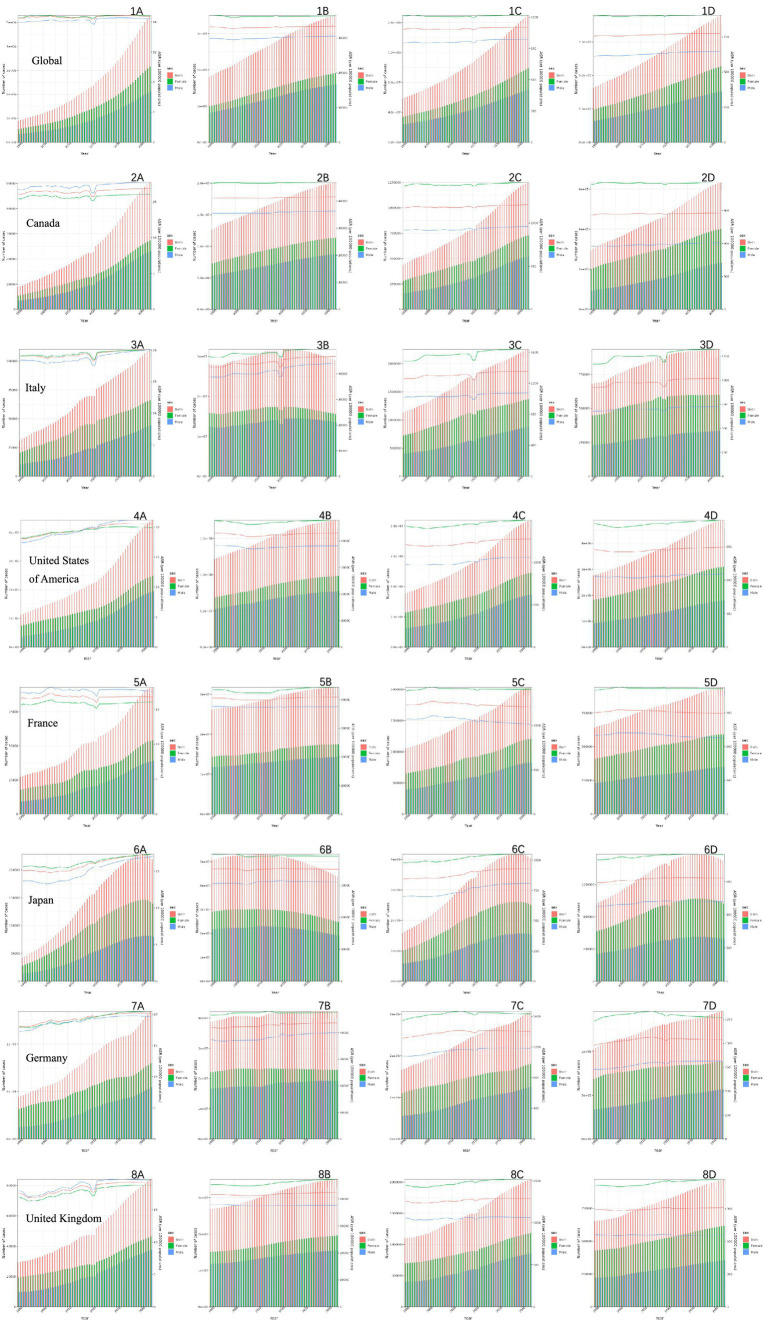
Forecast of neurological disease burden over the next 30 years using the Nordpred model. **(A)** Related death trends in different countries: observations and predictions using the Norpred APC model. **(B)** Related prevalence trends in different countries: observations and predictions using the Norpred APC model. **(C)** Related trends of DALYs in different countries: observations and predictions using the Norpred APC model. **(D)** Trends related to YLDs in different countries: observations and predictions using the Norpred APC model.

ASIR, Age-Standardized Incidence Rate; ASMR, Age-Standardized Mortality Rate; APC, Annual Percentage Change; AAPC, Annual Average Percentage Change;

Overall, based on predictions from the Nordpred age-period-cohort (APC) model for the next 30 years (Figure 5), the burden of neurological diseases in terms of deaths, cases, disability-adjusted life years, and years lived with disability is showing an upward trend in the analyzed countries. The total number of cases for deaths, illnesses, disability-adjusted life years, and years lived with disability is continuously increasing globally and in G7 member countries. This increase is observed in all regions, reflecting a steady rise in the disease burden over time. It is worth noting that there are significant gender differences in the number of cases, with females consistently having a higher proportion of deaths compared to males. However, in recent years, the gap between male and female deaths seems to have narrowed slightly, suggesting possible changes in disease dynamics or risk factors. In contrast, the age-standardized rate (ASR) per 100,000 population remains relatively stable during the forecast period. But in terms of predictions related to illnesses, we found that while the total number of cases is continuously increasing globally and in G7 member countries, the total number of cases in Japan is decreasing. Still, the standardized incidence rate remains relatively stable.

## Discussion

The Group of Seven, as representatives of developed countries, plays a crucial role in leading global public health efforts against health challenges in these regions. While each country within the G7 faces unique challenges, they also share common pressures, such as the dual burden of infectious and non-communicable diseases, healthcare disparities, and the need for more robust healthcare policies to address these issues (14).

Globally and within G7 member countries, there are significant gender disparities in the disease burden of neurological disorders. Data from 2021 shows that women bear a higher disease burden than men in most age groups, especially in terms of prevalence, which is consistent with previous research findings (10). This may be primarily due to the significant increase in migraine and tension-type headache attacks. Hormonal fluctuations, such as changes in estrogen and progesterone levels, can trigger headaches during menstrual cycles, pregnancy, and menopause in women (16, 17). Additionally, women are more susceptible to emotional stress in psychological and behavioral aspects, and early maladaptive schemas may also exacerbate headaches through psychological and physiological mechanisms (18). Sociocultural factors, such as women’s multiple roles in family and work, can lead to a high incidence of headaches due to chronic stress, and the lack of adequate social support may further exacerbate the problem (19). In terms of YLL, except for Japan and Italy, men bear a greater disease burden. This is broadly similar to the findings of Voskuhl R.R.’s study (20), which suggests that in multiple sclerosis, men with RRMS (relapsing–remitting multiple sclerosis) accumulate disabilities and progress to SPMS (secondary-progressive multiple sclerosis) significantly faster than women, and they also exhibit more severe cognitive impairment and gray matter atrophy compared to women. The significant gender disparities in the disease burden of neurological disorders reflect the complex interaction of biological, psychosocial, and environmental factors. In clinical practice, it is essential to consider gender as a specific factor and develop personalized diagnosis, treatment, and management strategies to improve patients’ quality of life and disease prognosis.

In terms of age distribution, neurological diseases are more common among people over the age of 10, which may be related to the development and maturation of the nervous system. The decreased neural plasticity and the excessive inflammatory response or immune system abnormalities brought on by the continuous maturation of immune function increase the risk of neurological diseases. The sharp rise in mortality rates after the age of 70 reflects the significant impact of aging on the burden of neurological diseases. As people age, the incidence of neurodegenerative diseases and cerebrovascular diseases increases significantly (21), and the incidence and severity of these diseases intensify with age. Aging not only increases the prevalence of these diseases but also exacerbates their impact on health, making it an important driver of the global disease burden (22). This trend indicates that the threat of aging to neurological health has become a major challenge in the field of public health, requiring prevention, early intervention, and treatment to reduce its burden. The burden of disease typically increases with age (7). Different situations can present different age patterns, emphasizing the need for targeted intervention measures and prevention strategies across the entire lifecycle.

Compared to global data, the burden of neurological diseases in G7 countries is relatively low, which may be attributed to their higher medical standards and more comprehensive public health systems. However, the number of DALYs in Japan and the United States is showing a gradual upward trend, which may be related to factors such as the epidemic situation of certain diseases and the allocation of medical resources in these countries. Japan is one of the countries with the most severe aging population in the world (23, 24), and the proportion of aging in the United States is also continuously rising. As people age, the incidence of neurological diseases such as Parkinson’s disease increases significantly (21), leading to a decline in patients’ quality of life and an increase in disability, thereby causing an increase in DALYs. Additionally, diabetic neuropathy is one of the fastest-growing neurological diseases, closely related to the high incidence of diabetes (25). The United States has a high prevalence of diabetes, while Japan, despite strict diabetes management, has seen an increase in obesity rates due to changes in dietary structure, indirectly driving the epidemic of neuropathy (10). Whether it’s the “big city centralization” of medical resource distribution in Japan (26) or the “medical-industrial complex” in the United States (27), both are market-oriented medical models. Remote areas and low-income populations, as the disadvantaged groups in medical resource allocation, often delay treatment due to cost issues, leading to an increased burden of disease.

Based on trend analysis from 1990 to 2021, the age-standardized incidence and mortality rates of neurological diseases globally have remained relatively stable overall, but there are significant differences among different countries and regions. For instance, the United States, Japan, and the United Kingdom experienced a downward trend in the incidence of neurological diseases from 1990 to 1999, while other G7 countries showed an upward trend. This variation could be attributed to different public health measures and medical policies adopted by each country during this period. For example, in the 1990s, the United States implemented the National Cholesterol Education Program (NCEP) (28) and the Joint National Committee on Prevention, Detection, Evaluation, and Treatment of High Blood Pressure (JNC) (29), integrating hypertension and hyperlipidemia management into the core public health agenda and reducing the risk of atherosclerosis-related strokes. Conversely, the DRGs payment system which effectively controlled medical costs but led to inadequate investment in prevention due to an overemphasis on treatment efficiency (30). Additionally, since 2015, the incidence trends for both men and women have increased globally, but G7 countries have shown opposite trends, indicating that these countries may have implemented more effective disease prevention and control measures.

When predicting the disease burden for the next 30 years using the Nordpred Age-Period-Cohort (APC) model, the burden of neurological diseases, including deaths, illnesses, disability-adjusted life years, and years lived with disability, is expected to rise globally and among G7 countries. This prediction suggests that despite progress in some areas, the overall burden of neurological diseases is still increasing and deserves sufficient attention. Notably, Japan has seen a decline in the number of prevalent cases, but the standardized incidence rate remains relatively stable. This might suggest advances in disease diagnosis and treatment in the country, but further research into specific etiologies and mechanisms is warranted.

To the best of our knowledge, this study marks the first-ever application of the Age-Period-Cohort model to scrutinize temporal patterns in the neurological disorder burden across developed nations, with a focus on G7 member states. This model offers unique insights into mortality risk variations between countries and significant trends within specific cohorts, enabling targeted recommendations through subsequent analyses. Our research findings hold potential to inform policymakers in crafting more precise interventions for distinct populations and offer invaluable insights for neurological disease prevention and control in less developed economies.

### Limitations

Our study faces several constraints. Primarily, the GBD 2021 dataset, compiled from diverse sources such as surveys, registries, and administrative records, exhibits significant disparities in data quality and comprehensiveness. This inconsistency introduces potential biases and uncertainties into our epidemiological estimations. Furthermore, the GBD framework heavily relies on modeling estimations for regions with limited empirical data. The assumptions inherent in these models may not be universally applicable, especially given the influence of cultural, genetic, and environmental factors on the neurological disease burden in G7 countries. Additionally, due to the five-year intervals of GBD 2021, our Age-Period-Cohort analysis spans multiple timeframes, which may obscure certain subtle variations in age, period, and cohort effects. Lastly, our study’s Age-Period-Cohort analysis relies on cross-sectional data estimated by GBD 2021, limiting our ability to determine precise risks at specific locations and times within G7 countries. We advocate for large-scale cohort studies to assess varying risks across diverse populations.

## Conclusion

This study presents a comprehensive examination of the neurological disease burden in developed countries, led by G7 member states. Our findings indicate a higher disease burden among females compared to males for most indicators. Neurological disorders are prevalent in individuals aged 10 and above, peaking between 25 and 39 years, with mortality rates escalating after 70 years. While the overall disease burden in the G7 remains lower than the global average, the United States and Japan exhibit a consistent upward trend in DALYs and mortality rates. Global age-standardized rates have remained steady between 1990 and 2021; however, male mortality rates have significantly risen in the United States, Japan, and Germany. Model projections suggest that due to population growth and aging, the overall number of neurological disease cases is expected to rise over the next 30 years, albeit with stable age-standardized rates. These findings underscore the pressing need for tailored intervention strategies that consider age, period, and cohort dimensions to effectively tackle the challenges posed by neurological disorders in developed nations. Moreover, they contribute valuable insights to neurological disease prevention and control efforts in developing countries.

## Data Availability

The original contributions presented in the study are included in the article/supplementary material, further inquiries can be directed to the corresponding authors.

## Footnotes

1
https://vizhub.healthdata.org/gbd-results/
